# Characterization of antibiotic resistance genes and mobile genetic elements in *Escherichia coli* isolated from captive black bears

**DOI:** 10.1038/s41598-024-52622-2

**Published:** 2024-02-02

**Authors:** Hang Liu, Keyun Shi, Yuhan Wang, Wenhao Zhong, Shulei Pan, Lei Zhou, Yuehong Cheng, Yu Yuan, Ziyao Zhou, Haifeng Liu, Shaqiu Zhang, Guangneng Peng, Qigui Yan, Yan Luo, Xiaoli Zhang, Zhijun Zhong

**Affiliations:** 1 College of Veterinary Medicine, Key Laboratory of Animal Disease and Human Health of Sichuan, Sichuan Agricultural University, Chengdu, 611130 China; 2 Jiangsu Yixing People’s Hospital, Yixing, 214200 China; 3 Sichuan Institute of Musk Deer Breeding, Dujiangyan, 611845 China; 4 Sichuan Wolong National Natural Reserve Administration Bureau, Wenchuan, 623006 China

**Keywords:** Molecular biology, Microbiology, Antimicrobials, Bacteria

## Abstract

The objective of this study was to analyze the antimicrobial resistance (AMR) characteristics produced by antibiotic resistance genes (ARGs), mobile genetic elements (MGEs) and gene cassettes in *Escherichia coli* isolated from the feces of captive black bears. Antimicrobial susceptibility testing was performed by using the disk diffusion method, and both MGEs and integron gene cassettes were detected by polymerase chain reaction. Our results showed that 43.7% (62/142) of the isolates were multidrug resistant strains and 97.9% (139/142) of the isolates were resistant to at least one antibiotic. The highest AMR phenotype was observed for tetracycline (79.6%, 113/142), followed by ampicillin (50.0%, 71/142), trimethoprim-sulfamethoxazole (43.7%, 62/142) and cefotaxime (35.9%, 51/142). However, all isolates were susceptible to tobramycin. *tetA* had the highest occurrence in 6 ARGs in 142 *E. coli* isolates (76.8%, 109/142). Ten mobile genetic elements were observed and *IS26* was dominant (88.0%, 125/142). *ISECP1* was positively associated with five β-lactam antibiotics. *ISCR3/14*, *IS1133* and *intI3* were not detected. Seventy-five *E. coli* isolates (65 *intI1*-positive isolates, 2 *intI2*-positive isolates and 8 *intI1* + *intI2-*positive isolates) carried integrons. Five gene cassettes (*dfrA1*, *aadA2*, *dfrA17-aadA5*, *aadA2-dfrA12* and *dfrA1-aadA1*) were identified in the *intI1*-positive isolates and 2 gene cassettes (*dfrA1-catB2-sat2-aadA1* and *dfrA1-catB2-sat1-aadA1*) were observed in the *intI2*-positive isolates. Monitoring of ARGs, MGEs and gene cassettes is important to understand the prevalence of AMR, which may help to introduce measures to prevent and control of AMR in *E. coli* for captive black bears.

## Introduction

Antimicrobial resistance (AMR) is a rapidly growing global health problem in animals and humans^1^. Antimicrobial resistance genes (ARGs) have been considered as a major mechanism of bacterial resistance to antibiotics^2^. Furthermore, the presence of bacteria carrying ARGs has been widely reported in wildlife in previous studies, which are major factors in the emergence of global health challenges^3^. Mobile genetic elements (MGEs), such as integrons, transposons and plasmids, play an important role in the spread of ARGs in animals and the environment^4^. Integrons, a type of MGE, can easily transfer one or more gene cassettes between different bacteria. Different gene cassettes that are incorporated into integrons have a significant correlation with resistance to different antibiotics^5,6^.

Black bear (*Ursus thibetanus*) is one of the national first-class protected wild animals in China, and there are more than 97 facilities are maintained for black bears and other bears in China^7,8^. There have been more reports on the epidemiological investigation of antibiotic resistant bacteria in captive wild animals^9–11^,and study for *E. coli* from sloth bear in India showed high prevalence of antimicrobial resistance, indicating that the AMR *E. coli* from captive black bears should be concerned^12^.Overuse of antibiotics has led to a problem of antibiotic resistant in clinical practice^13^. Previous studies have shown that the feces of healthy wild animals may serve as a reservoir for antibiotic resistant *Escherichia coli* (*E. coli*), which could pose a threat to public health and environmental safety^14,15^. Captive black bears have close contact with humans, including animal keepers and veterinarians. Exposure to antibiotic resistant *E. coli* from black bear feces may pose a potential risk to other animals and public health. To our knowledge, there is little information on the prevalence of AGRs, MGEs, integron gene cassettes, and the association between AGRs and MGEs in *E. coli* from captive black bears. The aim of this study was to characterize the antimicrobial resistance, especially the MGEs, ARGs and integron gene cassettes, of 142 *E. coli* isolates collected from the feces of captive black bears.

## Materials and methods

### Sample collection and bacterial strain identification

This study collected 142 fecal samples from a black bear breeding farm in Dujiang Yan city, China. Each sample was collected from one individual. Fresh fecal specimens (approximately 10 g) from each black bear were collected immediately by feeders after defecation on the ground and then quickly transferred into individual 50-mL plastic containers. All isolates (per isolate correspond to per sample) were identified using Gram staining, MacConkey (Solarbio, Beijing), and then confirmed by eosin methylene blue agar growth (Chromagar, France), and biochemical identification by API 20E system (BioMerieux, France)^16^. The verified and confirmed *E. coli* isolates were resuspended in tryptic soya broth plus 20% glycerol and stored at -20 ℃. A total of 142 strains were identified as DJY1-DJY142. DJY indicates the source of isolates from Dujiang Yan city, which is used in our lab for *E. coli* isolates from captive black bears.

### Analysis of antibiotic sensitivity and resistance patterns

Antimicrobial susceptibility was determined for all isolates against 13 antimicrobial agents via a standard disk diffusion test. The following antimicrobial disks (Oxoid) were used: aminoglycosides (gentamicin, GM, 10 μg; tobramycin, TOB, 10 μg), chloram phenicols (chloramphenicol, C, 30 μg), quinolones (ciprofloxacin, CIP, 5 μg), sulfonamide (sulfamethoxazole, RL, 25 μg), tetracyclines (tetracycline, TE, 30 μg), β-lactams (cephazolin, KZ, 30 μg; cefuroxime sodium, CXM, 30 μg; cefotaxime, CTX, 30 μg; cefepime, FEP, 30 μg; aztreonam, ATM, 30 μg; ampicillin, AMP, 10 μg; ampicillin/sulbactam 1:1, SAM, 10/10 μg). Results were interpreted based on the CLSI 2021 criteria. *E. coli* ATCC25922 was used as a control. The antibiotics used in this study were based on the information provided by the local farm veterinarians (CN, CIP, CTX, CXM and SAM were used for disease control); TOB, TE, C, KZ, FEP, CIP, SXT, ATM and AMP have been reported to be found antibiotic resistant in wildlife^17,18^.

### Screening for ARGs, MGEs and gene cassettes

Total genomic DNA was extracted using a kit (Tiangen Biotech, China). All genomic DNA solutions were stored at -20℃. According to previous studies, 6 antibiotic resistance genes (ARGs), 13 mobile genetic elements (MGEs) and gene cassettes were selected and detected by polymerase chain reaction (PCR)^19–22^. PCR assays were carried out in 25 μL volumes containing 2 μL template DNA, 12.5 μL 2 × Taq PCR Master Mix (Tsingke, China), 8.5 μL ddH_2_O (Solarbio, China) and 1 μL each primer. The PCR products were separated by gel electrophoresis in a 1.5% agarose gel stained with GoldView™ (Sangon Biotech, China), visualized under ultraviolet light and photographed using a gel documentation system (BioRad, USA). The primers and amplification conditions used have been previously described in Table S1^23,24^.

### Statistical analysis

All positive PCR products were directly sequenced in both directions by BGI (Beijing, China). Sequences were analyzed online by BLAST (http://blast.ncbi.nlm.nih.gov). *P*-values < 0.05 were considered to be statistically significant. The association between AMR phenotypes and the MGEs was calculated, and was considered significant at a *P*-value of < 0.05 and was reported as an odds ratio (OR) with 95% confidence interval (CI). An OR > 1 was considered a positive association or an increasing likelihood of co-occurrence of the MGEs or AMR phenotype, while an OR < 1 was considered a negative association. The statistical analyses were performed using the SPSS 27 software (StataCorp Lp, College Station, TX, USA).

### Ethics approval

This study was reviewed and approved by the Institutional Animal Care and Use Committee of Sichuan Agricultural University under permit number DYY-2020103018. Prior to the collection of fecal specimens from captive black bear, permission was obtained from the farm of black bear breeding, Dujiang Yan city, China.

## Results

### Antimicrobial susceptibility of 142 *E. coli* isolates

**Figure 1:**
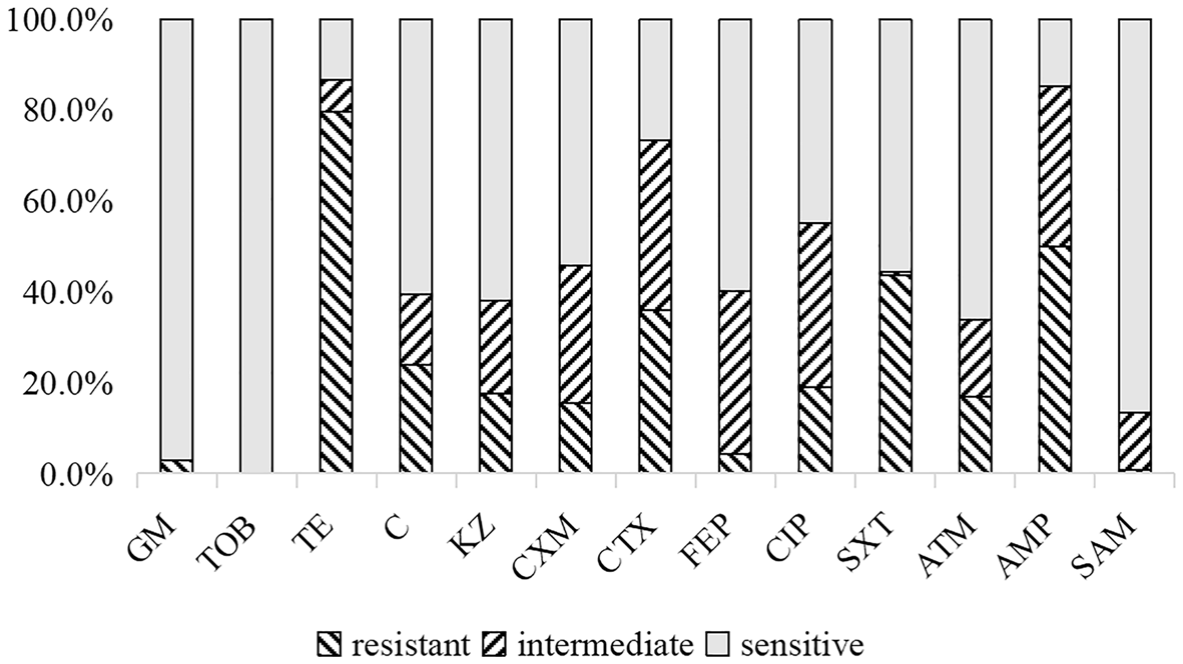
*E. coli* from black bears feces samples was resistant to various antibiotics. The highest resistance rate was TE (79.6%), followed by AMP (50.0%) and SXT (43.7%). All strains are sensitive to TOB.

**Figure 2:**
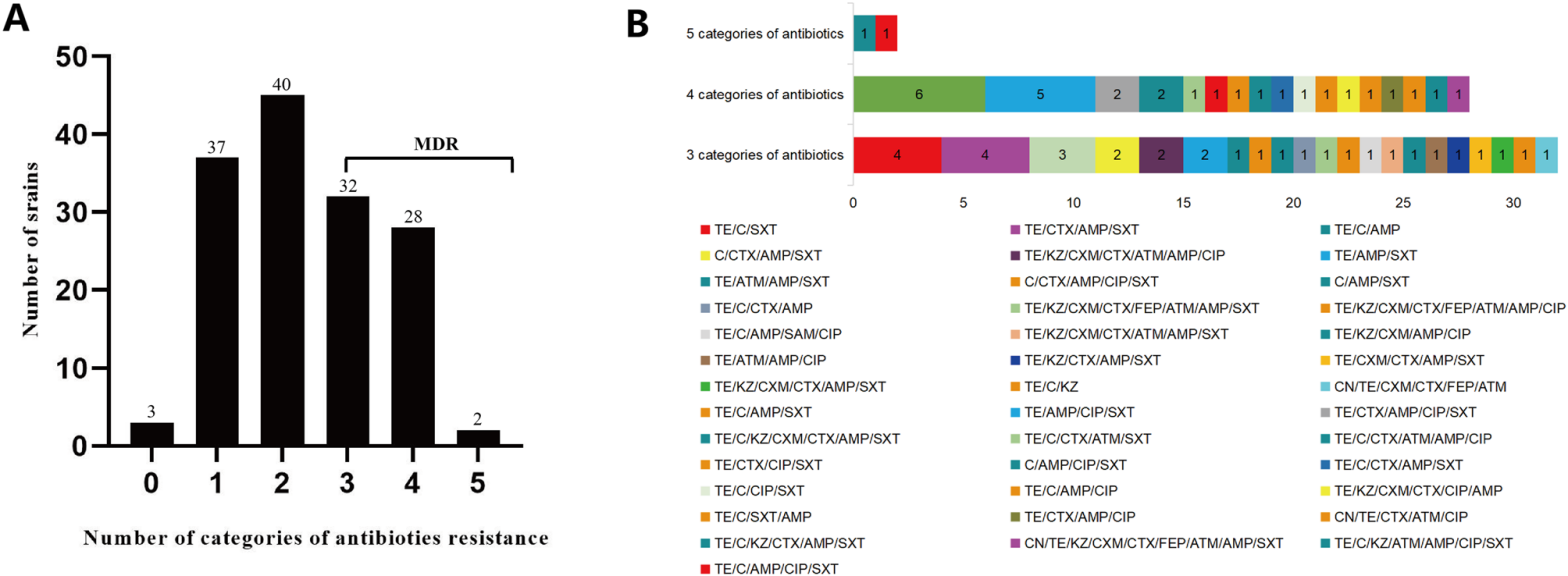
The resistant phenotype patterns of *E. coli* isolates from black bears. (**A**) The 0 on the x-axis means the strains were sensitive to all antibiotics tested and 1–5 means the strains resistant to 1–5 categories of antibiotics, respectively. The bars indicating that 62 *E. coli* isolates (62/142, 43.7%) are MDR. (**B**) Color bars demonstrating the distribution of resistant phenotype patterns in *E. coli* isolates from black bears and only MDR isolates were analyzed (n = 62). By using disk diffusion assay, a total of 40 resistant patterns were observed in MDR *E. coli* strains.

As shown in Table S2, a total of 142 *E. coli* isolates were obtained from the feces of 142 black bears in Sichuan, China. All *E. coli* strains showed resistance to the 13 antibiotics arranging from 0.0% (TOB) to 79.6% (TE) (Fig. 1). Of the142 strains, 139 strains (97.9%) were resistant to at least one of the 13 antibiotics tested. The most common resistances were to TE (79.6%, 113/142), followed by AMP (50.0%, 71/142) and SXT (43.7%, 62/142). Out of 142 strains, 62 strains (43.7%, 62/142) were found to be multidrug resistant (MDR), and 40 resistance patterns were observed in MDR strains (Fig. 2). Two strains (DJY14 and DJY43) were resistant to five classes of antibiotics, including: chloramphenicol, quinolone, sulfonamide, tetracycline and β-lactam. A total of 70 antibiotic resistance patterns were observed, with the three most prevalent patterns were TE (17.6%, 25 isolates), TE/SXT (7.0%, 10 isolates) and TE/C/AMP/SXT (4.2%, 6 isolates). In particular, the DJY127 strain was resistant to nine antibiotics (CN/TE/KZ/CXM/CTX/FEP/ATM/AMP/SXT).

### Identification and characterization of ARGs from 142 *E. coli* isolates

As shown in Table S2, 5 ARGs were detected in 142 *E. coli: tetA* (70.8%, 109/142), *qnrS* (35.2%, 50/142), *flor* (23.9%, 34/142), *sul1* (25.4%, 36/142) and *bla*_*CTX-M*_ (12.7%, 18/142). The aminoglycosides resistant gene *aphA3* was not detected in 142 *E. coli.* A total of 125/142 (88.0%) strains carried ARGs, with *tetA* being the most common pattern (36/142, 25.4%). Among the 142 *E. coli* isolates, 39 isolates (27.5%, 39/142) carried more than three types of ARGs. We further analyzed the concordance rate of ARGs and AMR detected in 142 *E. coli* strains. The results showed that 18.0% to 88.9% of ARGs corresponded to antibiotics resistance phenotypes (Table 1).

**Table 1:** Distribution of ARGs and AMR detected in 142 *E. coli* strains.

AGR	Number of positive isolates (n)	Strains with corresponding AMR (n)	Recombination rate (%)
*tetA*	109	94	86.2
*qnrS*	50	9	18.0
*sul1*	36	22	61.1
*flor*	34	21	61.8
*bla*_*CTX-M*_	18	16	88.9
*aphA3*	0	0	–

### Mobile genetic elements prevalence in 142 *E. coli*

**Figure 3:**
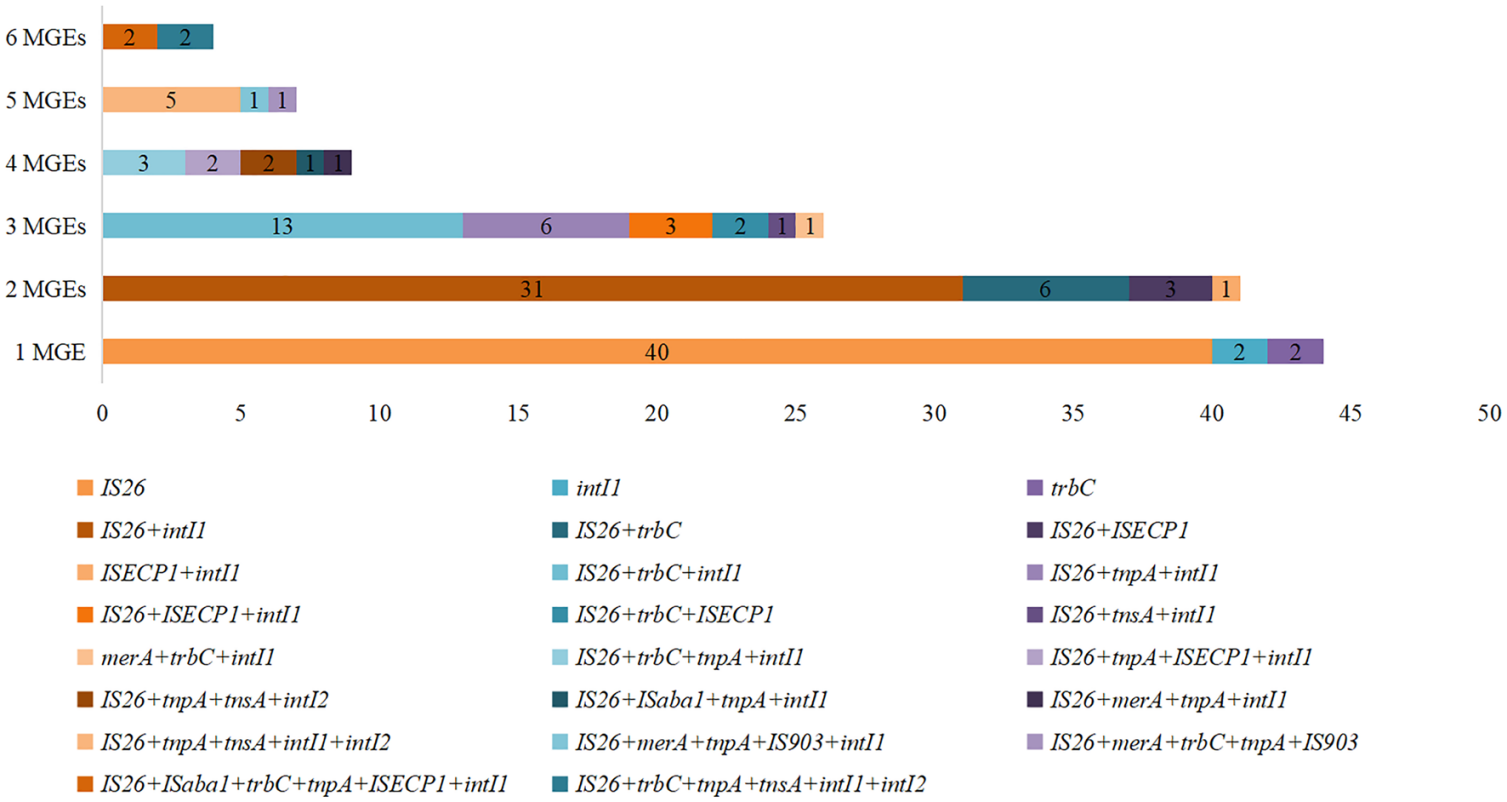
Components of mobile genetic elements in 142 *E. coli* isolates from black bears. Color bars demonstrating the distribution of MGEs combination patterns in *E. coli* isolates from black bears, a total of 23 MGEs combination patterns were observed in *E. coli* strains.

Ten out of the 13 MGEs were detected in the 142 isolates: *IS26* (88.0%, 125/ 142), *intI1* (52.8%, 75/142), *trbC* (22.5%, 32/142), *tnpA/Tn21* (18.3%, 26/142), *ISECP1* (9.2%, 13/142), *tnsA* (7.0%, 10/142), *intI2* (6.3%, 9/142), *merA* (2.8%, 4/142), *Isaba1* (2.1%, 4/142) and *IS903* (1.4%, 2/142).However, *IntI3*, *ISCR3/14* and *IS1133* were not detected in any of the 142 *E. coli*. There were 23 patterns of combinations among 13 MGEs (Fig. 3), in which the *IS26* was predominant (28.1%, 40/142), followed by *IS26* + *intI1* (21.8%, 31/142) and *IS26* + *trbC* + *intI1* (7.7%, 11/142). No mobile genetic element was detected in eleven isolates (DJY18, DJY36, DJY41, DJY44, DJY47, DJY56, DJY70, DJY80, DJY102, DJY103 and DJY119).

### Characterization of integrons and gene cassettes in 142 *E. coli* isolates

As shown in Fig. 3, 52.8% (75/142) and 6.3% (9/142) of isolates were found to be positive for *intI1* and *intI2*, respectively. *intI1* and *intI2* were both detected in six strains (DJY127, DJY130, DJY136, DJY140, DJY141 and DJY142). None of the 142 isolates tested positive for *intI3* , and 45.1% (64/142) of isolates were negative for *intI1* or *intI2*. Gene cassettes were also analyzed in *intI1/2* positive isolates. As shown in Table 2, among 75 *intI1-*positive isolates, we detected 5 different gene cassette arrays:*dfrA1-aadA1*(1 isolate), *dfrA1*(2 isolates), *dfrA17-aadA5*,(4 isolates), *aadA2*(5 isolates), *aadA2-dfrA12* (11 isolates) in 23 *E. coli* isolates. Among the 9 *intI2-*positive isolates, 6 *E. coli* isolates had 2 gene cassette arrays, *dfrA1-catB2-sat1-aadA1*(1 isolate), *dfrA1-catB2-sat2-aadA1*(5 isolates) arrays in 6 *E. coli* isolates.

**Table 2:** Gene cassettes among 72 integron positive *E. coli* isolates from black bear.

Class 1 integron positive strains	Gene cassttes	Class 2 integron positive strains	Gene cassttes
DJY3,DJY10,DJY13,DJY14,DJY15,DJY22,DJY37,DJY48,DJY49,DJY55,DJY58,DJY59,DJY63,DJY66,DJY67,DJY68,DJY69,DJY72,DJY76,DJY81,DJY82,DJY84,DJY85,DJY88,DJY89,DJY90,DJY91,DJY92,DJY93,DJY95,DJY96,DJY99,DJY100,DJY101,DJY105,DJY107,DJY109,DJY112,DJY114,DJY115,DJY117,DJY122,DJY123,DJY125,DJY127,DJY128,DJY130,DJY135,DJY136,DJY138,DJY139	–	DJY140, DJY141, DJY142	–
DJY108	*dfrA1-aadA1*	DJY134	*dfrA1-catB2-sat1-aadA1*
DJY17, DJY62	*dfrA 1*	DJY123, DJY126, DJY127, DJY130, DJY136	*dfrA1-catB2-sat2-aadA1*
DJY45, DJY46, DJY97, DJY98	*dfrA17-aadA5*		
DJY94, DJY137, DJY140, DJY110, DJY113	*aadA2*		
DJY57, DJY60, DJY71, DJY73, DJY74, DJY83, DJY87, DJY116, DJY131, DJY141, DJY142	*aadA2-dfrA12*		

“–”: Gene cassettes not detected in this study.

### Association among AMR phenotypes and MGEs

For the associations between AMR phenotypes and MGE, 14 pairs were positively associated (OR > 1) and 1 pair was negatively associated (ATM/*IS26*, OR, 0.308) (OR < 1) (Table 3). The strongest positive associations were observed between CN and *tnpA* (OR, 15.000; 95% CI 1.493–150.668), followed by CXM/*ISECP1* (OR, 13.143; 95% CI 3.775–45.759) and FEP/*ISECP1* (OR, 12.600; 95% CI 2.245–70.717). It is worth noting that *ISECP1* was found be positively associated with five β-lactam antibiotics (CXM, CTX, FEP, ATM and AMP). No associations were observed for *intI3, ISCR3/14* and *IS1133* with 13 antibiotics.

**Table 3:** The associations between MGEs and AMR among *E. coli* isolates from black bear in Sichuan, China (n = 142).

	IS26(125)	tbrC(32)	tnpA(26)	ISECP1(13)	tnsA(10)	intI1(75)	intI2(9)
GM (4)	NA	11.276 (1.131–112.459)	15.000 (1.493–150.668)	NA	–	NA	–
KZ (109)	–	3.625 (1.028–12.787)	4.376 (0.977–19.613)	–	–	NA	–
CXM (22)	NA	–	5.417 (2.011–14.586)	13.143 (3.775–45.759)	4.222 (1.084–16.439)	–	5.111 (1.253–20.841)
CTX (51)	–	–	–	12.238 (2.592–57.780)	–	–	–
FEP (6)	NA	–	–	12.600 (2.245–70.717)	–	–	–
SXT (62)	–	–	–	–	–	2.641 (1.328–5.255)	–
ATM (24)	0.308 (0.101–0.939)	–	–	11.300 (3.290–38.812)	–	–	–
AMP (71)	5.567 (1.524–20.340)	–	–	3.716 (0.977–14.130)	–	–	–

*Only significant association (*P* < 0.05) are shown.

µ Odds ratio (OR) for significant associations between MGEs and AMR (95% confidence interval in parenthesis); NA, no results available (or) could not be calculated because none of the isolates carried one of the pairs of MGEs and AMR or one of the rate of detected was zero).

– indicates no significant associations (*P* > 0.05).

## Discussion

To enhance comprehension of the AMR of *E. coli* isolates from captive black bears, we assessed 142 *E. coli* strains from 142 captive black bears. Of these strains, 43.7% exhibited multidrug resistance (MDR)*.* The MDR rate of *E. coli* in black bears is similar to that of sloth bear (51.1%) and giant panda (43.4%)^24,25^, but lower than that of domestic animals and poultry^26^. Antibiotics have been widely applied to promote growth and prevent diseases in China, which may lead to high MDR strains detected in domestic animals and captive wild animals^27–29^. Our study found a high rate of antibiotic resistance in *E. coli* isolates (97.9%) from black bears, which is higher than that observed in sloth bears in India (93.3%)^25^.The consumption and production of antibiotics are higher in China^30^, and the frequent exposure or misuse of antimicrobials in animals may contribute to the emergence and spread of resistance in *E. coli* from black bears. In our study, *E. coli* from black bear exhibited varying degrees of resistance to GM, CIP, CTX and CXM. GM, CIP, CTX and CXM have been used for disease control in black bears, which may contribute to antimicrobial resistance. Additionally, the resistance rate to TE (79.6%) in *E. coli* is the highest rate among 13 antibiotics^31^. The high resistance rate to TE (79.6%) has also been reported in sloth bears (51.1%)^25^. TE has been used to treat animal infections in the world^32^, however, the development of resistance has narrowed their utility and the use of TE is strictly regulated in China^33^. Our previous study was also found this phenomenon in giant panda, indicating that AMR persists for a longer time and is not easy to eliminate^34^. In general, ARGs were the primary cause of antibiotic resistance phenotypes, AGRs were expressed and translated into proteins, allowing bacteria to achieve resistance to antibiotics. Different ARGs mediate bacterial resistance to antibiotics in different ways^35^. In our study, 6 ARGs were detected and the most prevalent ARG was *tetA* (70.8%, 109/142). The high prevalence of *tetA* has also been observed in various wild animals (e.g., giant panda, monkey) and human studies^23,24,36^, suggesting that TE resistance will continue to exist for a period of time, and we should continue to monitor TE resistance. Moreover, we also found that some isolates (DJY62 and DJY129) showed antimicrobial susceptibility despite harboring ARGs. This phenomenon may be related to abnormal expression of ARGs, or the expression of ARGs may not have reached the level required to produce resistance^37^.

Previous studies have shown that MGEs play an important role in the dissemination of ARGs^38,39^. Our result showed that 10 MGEs and 23 MGEs combination patterns were detected in 142 *E. coli* strains from black bears. In comparison to our previous research, we found fewer MGEs (10) and MGEs combinations (23) in black bears than in giant pandas (11 MGEs, 35 MGEs combinations)^24^. We detected 10 *bla*_*CTX-M*_ positive strains out of 13 strains carrying *ISECP1* (Table S2). Studies have shown that *ISECP1* and *bla*_*CTX-M*_ are all located in the same plasmid, which may be the reason for their association with five β-lactams antibiotics^40^. Horizontal transfer of ARGs mediated by MGEs is one of the main mechanisms^41,42^, and it is necessary to regularly monitor MGEs in *E. coli* from black bears. To our knowledge, this study is the first to examine MGEs of *E. coli* isolates from captive black bears.

Integrons, as one of the MGEs, are known to capture gene cassettes, which could be transferable among bacteria and disseminate ARGs via transmissible plasmids and insertion sequences, posing a threat to public health^16,43^. In our results, class 1 integron (56.8%) was more prevalent compared to class 2 integron (6.3%), while class 3 integron was not detected, which is consistent with results from yaks, ducks and giant pandas^16,24,44^. Research has shown that due to the influence of human activities, the prevalence of Class I integron in natural environments is higher than that of Class II and III^45^. Gene cassettes harbored in integron-positive isolates are an important medium for the spread of ARGs^46^. In this study, seven types of gene cassettes were detected in class 1 or class 2 integron-positive isolates (Table 2). The gene cassette encoding the aminoglycoside adenyl transferase *(aadA2*) and dihydrofolate reductase (*dfrA12*) 34 is the most frequently detected among the isolates, with 11 instances^34^. Similar gene cassettes belonging to *dfrA*, *sat*, *catB* and *aadA* have also been detected in *E. coli* isolates from other animals, including giant pandas and rabbits^24,47^, indicating that the gene cassettes have spread between different species.

The above results indicate that different types of integrons and gene cassettes are carried by *E. coli* from captive black bears, posing a threat of spreading ARGs to other animals or environments. The high prevalence of AMR *E. coli* detected in black bears pose a potential risk to public health. Therefore, relevant people like veterinarians should pay attention during work. The origin of the AMR isolates analyzed in our present study is unclear, despite the high prevalence of AMR *E. coli* detected within the farm-raised black bear population.

## Conclusion

This study found a high prevalence of antibiotic resistance and a diverse range of MGEs observed among the *E. coli* strains isolated from captive black bears. A positive association was observed between MGEs and AMR in *E. coli* strains. It is essential to monitor the distribution of ARGs, MGEs and gene cassettes, which may help to introduce interventions for the prevention and control of AMR in *E. coli* among captive black bears.

### Supplementary Information

Supplementary Information 1.

Supplementary Information 2.

## Data Availability

The datasets generated and/or analyzed during the current study are available from the corresponding author on reasonable request.
